# Unmet Needs of People With Parkinson's Disease and Their Caregivers During COVID-19-Related Confinement: An Explorative Secondary Data Analysis

**DOI:** 10.3389/fneur.2020.615172

**Published:** 2021-01-18

**Authors:** Anne-Marie Hanff, Claire Pauly, Laure Pauly, Valerie E. Schröder, Maxime Hansen, Guilherme Ramos Meyers, Anne Kaysen, Linda Hansen, Femke Wauters, Rejko Krüger

**Affiliations:** ^1^Transversal Translational Medicine, Luxembourg Institute of Health, Strassen, Luxembourg; ^2^Parkinson Research Clinic, Centre Hospitalier de Luxembourg, Luxembourg, Luxembourg; ^3^Translational Neuroscience, Luxembourg Centre for Systems Biomedicine, University of Luxembourg, Esch-Belval, Luxembourg

**Keywords:** COVID, needs assessment, health services needs and demand, Parkinson disease, pandemic, qualitative research

## Abstract

Self-perceived unmet needs in people with typical and atypical parkinsonism (PwP) and their caregivers, support network, personalized ways to address self-perceived unmet needs during confinement, as well as the prevalence of self-reported COVID-19 related symptoms, confirmed SARS-CoV-2 infection, and self-reported COVID-19 related hospitalization in Luxembourg and the Greater Region were assessed. From 18th March to 10th April 2020, 679 PwP were contacted by phone. Data was collected in the form of a semi-structured interview. The thematic synthesis identified 25 themes where PwP need to be supported in order to cope with consequences of the pandemic, and to adapt their daily and health-related activities. The present work highlights that in the context of personalized medicine, depending on the individual needs of support of the patient the identified self-perceived unmet needs were addressed in various ways ranging from one-directed information over interaction up to proactive counseling and monitoring. Family and health professionals, but also other support systems were taking care of the unmet needs of PwP (e.g., shopping, picking-up medication, etc.) during the pandemic. 7/606 PwP (1.15%) reported COVID-19 related symptoms, 4/606 (0.66%) underwent a rRT-PCR-based diagnostic test and 2/606 (0.33%) were confirmed as SARS-CoV-2 positive. None of these PwP reported being hospitalized due to COVID-19. Our results will allow health professionals to expand their services in a meaningful way i.e., personalize their support in the identified themes and thus improve the healthcare of PwP in times of crisis.

## Introduction

The outbreak of COVID-19 was declared a global pandemic by the WHO in March 2020. As a result, many countries, including Luxembourg and the Greater Region, introduced restrictions and recommendations to prevent the spread of the virus emphasizing the urgency to adhere to social isolation and social distancing. These factors have profoundly changed people's daily routines over a short period of time and especially for people with an underlying chronic illness such as Parkinson's Disease (PD) (1).

Previous work by Prasad, Holla (2) focused on the perceptions and implications of COVID-19 in PwP and their caregivers. New problems attributed to the pandemic were reported and associated with loss of access to healthcare and medication. Additionally, worsening of extrapyramidal symptoms or appearance of new symptoms was reported by patients and healthcare professionals. Schirinzi et al. (3) analyzed 162 E-mails, phone texts and phone vocal messages spontaneously sent from PwP or caregivers to the PD Clinic. Queries and communications were classified in four groups depending on the content: relationship between COVID-19 and PD; acute changes in neurological symptoms; occurrence of intercurrent medical/surgical conditions and clinical services. As a limitation, the authors report that their work is not a systematic collection of information about self-perceived unmet needs in a PD population. Furthermore, the applied methodology of classification wasn't specified limiting the interpretation and the reproduction of the results.

Based on the nation-wide cohort of PwP recruited within the Luxembourg Parkinson's study (4) we were in a unique position to address the important issue of self-perceived unmet needs and the situation of PwP and their caregivers in Luxembourg and the Greater Region during COVID-19-related confinement.

Our study aimed at exploring the diversity of unmet needs of PwP and their caregivers during COVID-19-related confinement. Moreover, we investigated the reported support networks during confinement and personalized addressing of self-perceived unmet needs. Additionally, we assessed the frequency of self-reported COVID-19 related symptoms (i.e., fever, cough and/or respiratory distress), confirmed SARS-CoV-2 positive cases, and self-reported COVID-19 related hospitalization.

## Method

Methods and findings are reported according to the reporting guideline COREQ (5).

In the frame of the Luxembourg Parkinson's study (4), participants of a cohort approved by the National Ethics Board (CNER Ref: 201407/13) were contacted by phone from 18th March to 10th April 2020, starting 2 days after implementation of confinement in Luxembourg. Overall, 679 PwP were eligible for being contacted by phone (resident in Luxembourg and the Greater Region, capable to participate). Consequently, the collection of diverse perspectives was allowed. Five hundred seventy-four of 679 (84.5%) were diagnosed with typical PD. PwP that were not reached after three contact attempts per phone and a contact attempt via email were classified as “not reachable.”

The initial aim of the phone calls was to evaluate and ensure the provision of care for PwP during COVID-19-related confinement. To assess the presence of unmet needs, PwP and their family members were asked, whether they experienced a lack of care and who takes care of them during the confinement. Unmet needs were defined as the absence of diagnostic or therapeutic alternatives (6). COVID-19 related information was collected by asking the following questions: Do you suffer from COVID-19 related symptoms (i.e., fever, cough and/or respiratory distress)? If yes, did you get a COVID-19 test? If yes, did you get a positive COVID-19 result? If yes, were you hospitalized because of the COVID-19 infection? Data was collected by an interdisciplinary team of secretaries, project managers, nurses, medical doctors, and neuropsychologists in the form of a semi-structured interview. Nine interviewers were female and two were male. Most interviewers had experience in the conduction of phone calls and were already in contact with the PwP/their caregivers in the frame of the telephone questionnaires of the Luxembourg Parkinson Study and the annual follow-up visits. No further characteristics about the interviewer (bias, assumptions, reasons and interest in the research topic) were documented.

The semi-structured interviews were neither recorded, nor transcripted. The authors expected interview notes to allow the descriptive exploration. The interview notes provided no information about the duration or the repetition of the interviews. No quantitative hypothesis was tested. The project was considered as explorative secondary data analysis (7).

**Figure 1:**
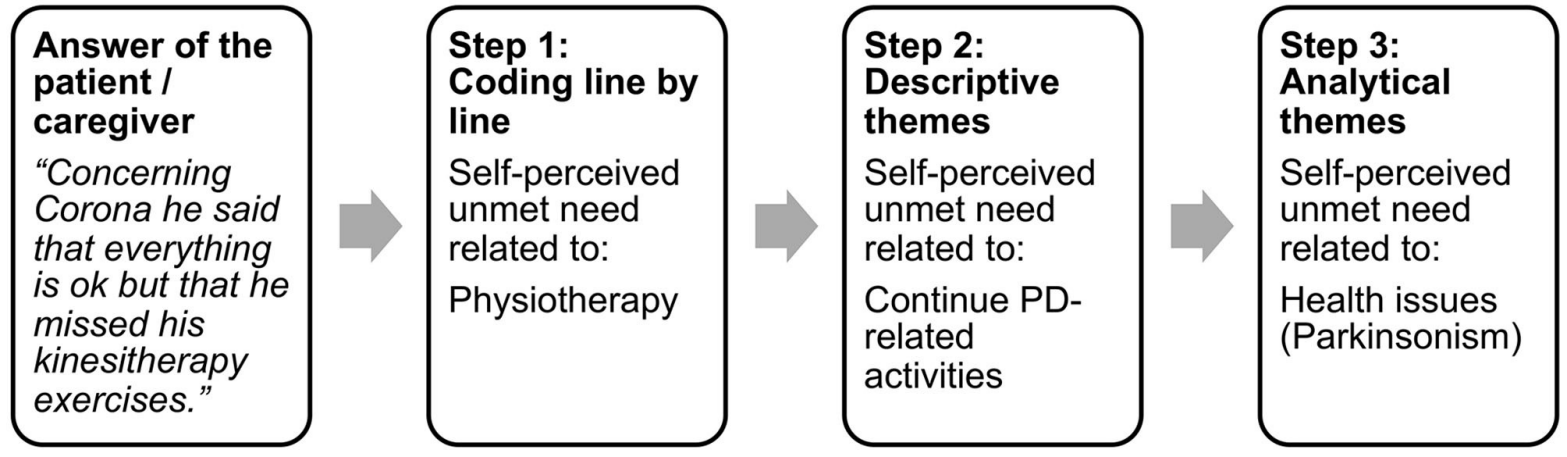
Exemplar process from “coding line by line” to “analytical themes”.

Secondary data e.g., interview notes of PwP' and/or their caregivers' anonymous answers were analyzed by “thematic synthesis” (8). The method of “thematic synthesis” was chosen to systematically organize data into a structured format. Following questions were guiding the analysis: What unmet needs did PwP or their caregivers report? What support network did they mention? What interventions were offered by the clinical team to what kind of patients? Figure 1 illustrates the method of the “thematic synthesis” i.e., the process from “coding line by line” to “analytical themes.” In the first step, four team members independently coded the answers of the patients i.e., defined line by line the keywords describing the meaning and content of the semi-structured interviews. In the second step, the team members looked for similarities and differences between the defined keywords. Similar self-perceived unmet needs were grouped, named by a descriptive theme and this process resulted in a hierarchical tree structure with four layers (emotional distress, alternative ways to continue daily activities, COVID-19, parkinsonism) to organize a total of 25 descriptive themes illustrated in Table 1. In a third step, the “analytical themes” (Consequences of the pandemic situation and health issues) were generated. These represented a stage of interpretation whereby the clinical team “went beyond” the primary notes and generated new conceptions via group discussions. The 25 descriptive themes and the four layers were examined again in light of these constructs and changes were made accordingly. This cyclic process was repeated until the two analytical themes were able to describe each of the initially reported self-perceived unmet needs. A figure illustrating the coding tree is provided as Supplementary Material 1. The same method was applied for the identification of the different ways to address unmet needs and support network during confinement. After analyzing the interview notes, no relevant knowledge was obtained from new participants and data saturation was reached.

**Table 1:** Self-perceived unmet needs of people with parkinsonism and their caregivers during COVID-19 related confinement were related to the following themes.

**Consequences of the pandemic situation**	
**Emotional distress**	**Alternative ways to continue daily activities**
Risk of infection with COVID-19 Physical distancing Restricted communication with family and friends Non-adherence to hygiene recommendations by health care professionals Administrative issues (finances, insurance,…) Illness and/ or death of a family member	Rescheduling private appointments (doctors, therapists, vacation,…) Transformed health system Communication with family and friends Shopping (food, hygiene articles,…) Hobbies (meeting with the music group,…)
**Health Issues**	
**COVID-19**	**Parkinsonism**
General recommendations Protection of vulnerable persons (living in the same household) Procedure in case of contact with COVID-19 positive person Survey of COVID-19 symptoms In the case of COVID-19 symptoms: Referral to a health professional	Vulnerability of PwP Interaction of PD-therapy with COVID-19 Reduced effectiveness of PD-medication due to reduced physical activity Availability of PD medication
	**Continue PD-related activities:**
	Parkinson Association Physiotherapy Prescription of PD medication
	**Unmet needs not related to COVID-19:**
	PD-related complications Treatment-related complications

Descriptive statistics were performed on data covering COVID-19 related symptoms, patient-reported Real-time Reverse-Transcriptase-Polymerase Chain Reaction (rRT-PCR)-confirmed SARS-CoV-2-positive cases, related hospitalizations, and self-perceived unmet needs. SPSS Statistics version 25 was used, all tests were two-sided and *p*-values of ≤ 0.05 were considered statistically significant.

## Results

We successfully contacted 89.25% (606/679) of the eligible PwP participating in the Luxembourg Parkinson's study (4). Descriptive statistics showed the mean age was 67.22 years (SD = 10.32), mean accomplished years of education was 12.91 years (SD = 1.13) and one third (205/606, 33.83%) of the respondents were female. To check for a potential bias due to PwP “not reachable via phone” (7, 9) a subgroup analysis was conducted comparing both groups' characteristics of PwP that were successfully contacted (*n* = 606) with those that were not reached (*n* = 73). The independent-sample *t*-test showed no significant differences in the demographic variables current age, years of education, and gender (*p* > 0.05).

In total 25 unmet needs were explored. Self-perceived unmet needs detailed in Table 1 were either related to the consequences of the pandemic situation (emotional distress and alternative ways to continue daily activities) or to health issues (COVID-19 and parkinsonism). Of note, self-perceived unmet needs unrelated to COVID-19 e.g., typical PD symptoms or side-effects of PD medication remained important.

The Supplementary Materials 2, 3 describes the analytical themes more in detail, and provides examples of interview notes.

**Figure 2:**
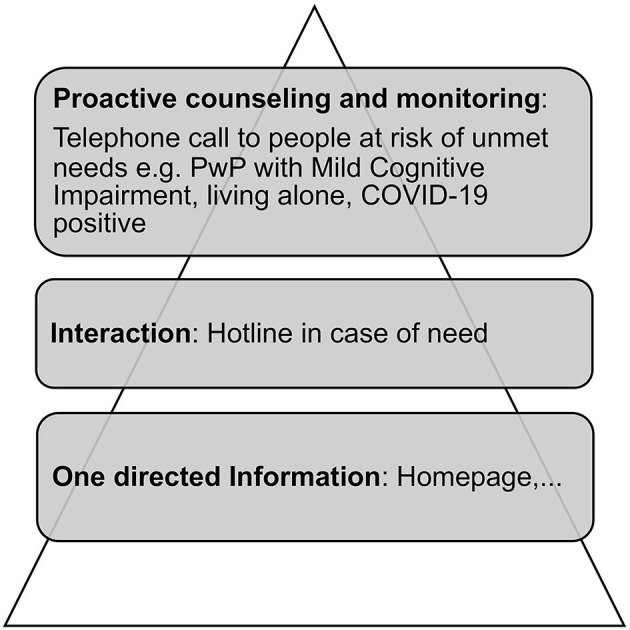
Personalized addressing of self-perceived unmet needs.

As illustrated (Figure 2), the thematic synthesis highlighted that in the context of personalized medicine, depending on the individual needs of patient's support, the identified self-perceived unmet needs were addressed in various ways ranging from one-directed information over interaction up to proactive counseling and monitoring.

**Figure 3:**
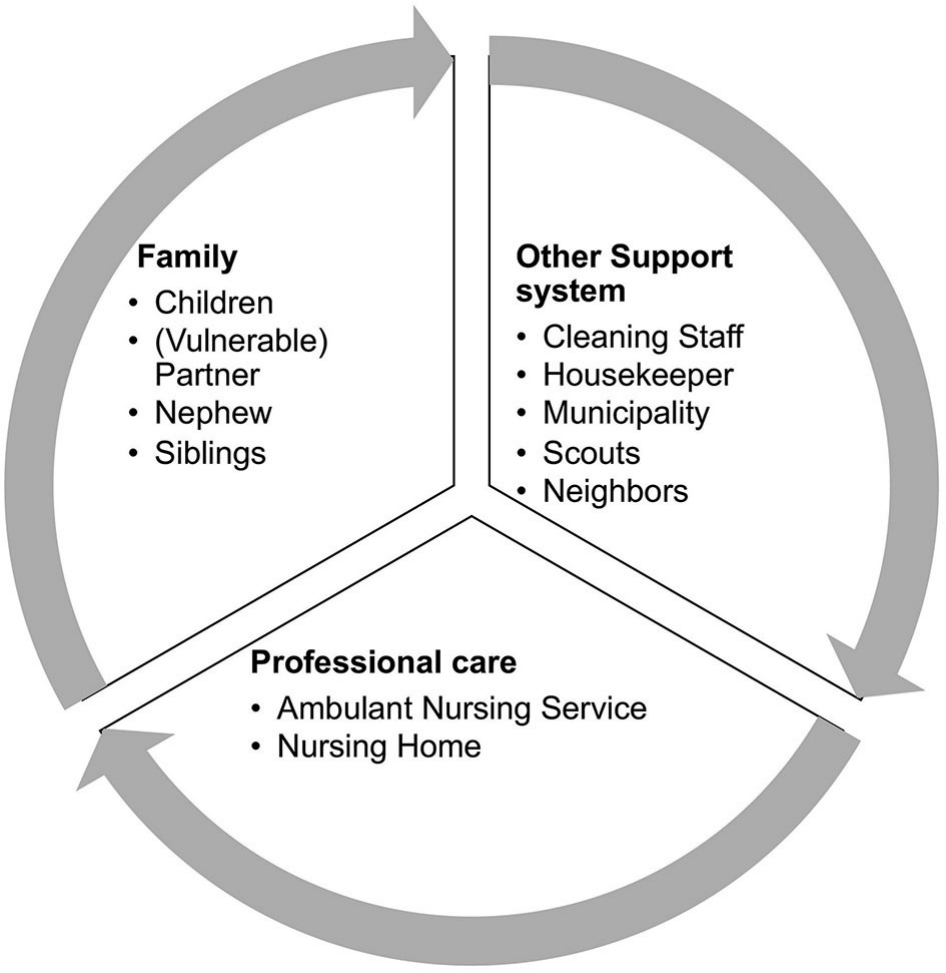
Reported support network during confinement.

The thematic synthesis revealed that the family and health professionals, but also other support systems were taking care of the unmet needs of PwP (e.g., shopping, pick-up of medication, etc.) during the pandemic (Figure 3).

Overall 7/606 PwP (1.15%) reported COVID-19 related symptoms, 4/606 (0.66%) underwent an rRT-PCR-based diagnostic test and 2/606 (0.33%) were confirmed as SARS-CoV-2 positive. None of these PwP reported a needed hospitalization due to COVID-19.

## Discussion

Our study assessed the self-perceived unmet needs of PwP and their caregivers during COVID-19-related confinement on a national level. Our observations indicate that, an increased support in the identified themes is necessary to cope with the consequences of the pandemic, i.e., the emotional distress, and to adapt their daily and health-related activities.

Unmet needs related to the consequences of the pandemic situation were explored and described for the first time. Whereas, Schirinzi et al. (3) analyzed incoming calls, the present work was based on proactively calling eligible participants of the Luxembourg Parkinson's Study. In contrast to this previous work on unmet needs in PwP, which classified queries and communications without specifying the applied methodology limiting the interpretation and the reproduction of the results, the applied thematic synthesis allowed us to “go beyond” the primary information and to transparently generate new conceptions (consequences of the pandemic situation and health issues). Our work confirms the results of Schirinzi et al. (3) having identified unmet needs related to health issues (e.g., relationship between COVID-19 and PD; acute changes in neurological symptoms; occurrence of intercurrent medical/surgical conditions; clinical services).

Participants of the present study reported the necessity to reschedule appointments with their neurologist, although PwP are in need of regular visits because of symptoms' progression and adjustment of their medication (2). E-Health solutions as described by Miele and colleagues (10) should be considered to ensure patient needs and continuity of care even in times such as the COVID-19 pandemic.

Another very important aspect of the pandemic is the increased reduction of physical activity. With the restrictions in place, people find themselves stuck at home without any possibility to go outside or to follow sessions with their physiotherapist (11). Recent findings (12, 13) have shown that physical exercise may attenuate clinical symptom progression in PD and a loss of exercise results in a worsening of the motor symptoms in PD. Additionally, a lack of physical activity can increase non-motor symptoms such as insomnia, constipation and could lead to psychological stress which, in return, also aggravates symptoms of PD (14). Our findings confirm previous results as participants reported unmet needs related to the inability to continue physiotherapy along with the consequences of reduced physical activity.

During confinement, the number of hours of caregiving increases dramatically, and as expected, our study identified family members as part of the reported support network. Consequently, caregiver burden presumably increases during confinement. Mosley, Moodie (15) summarized symptoms of PwP (e.g., motor and neuropsychiatric symptoms) associated with caregiver burden. Caregivers of PwP reporting such symptoms during confinement should get proactive counseling and monitoring to reduce the risk of caregiver burnout, and prevent premature institutionalization of PwP.

The described personalized addressing of self-perceived unmet needs points out the importance of an individual approach in patient information, interaction, proactive counseling, and monitoring. The intensity of interactions increases with patient complexity. Peek and Baird (16) defined patient complexity as interference with standard care and decision-making by symptom severity or impairments, diagnostic uncertainty, difficulty engaging care, lack of social safety or participation, disorganization of care, and difficult patient-clinician relationships. In the present work, PwP with mild cognitive impairment, living alone or being COVID-19 positive could be considered as complex patients. This group of PwP at risk of unmet needs were contacted proactively to prevent complications resulting from the confinement. These findings help to develop personalized interventions for PwP during confinement.

The numbers of PwP reporting a SARS-CoV-2 infection corresponds to the prevalence in the general Luxembourgish population (17). However, these numbers must be interpreted with caution, as this study was conducted at the very beginning of the confinement. For this reason, the data does not allow conclusions to be drawn about the vulnerability of PwP.

In this explorative design, the qualitative method of data analysis was a valuable alternative to traditional quantitative methods as data was available in the form of a semi-structured interview. Implicit information i.e., unquantifiable patterns had to be observed first and only then, generalizations based on the observations could be made. To our knowledge, implicit information couldn't have been extracted by the traditional quantitative methods (9). The large number of participants as well combination of the quantitative and qualitative approaches helped to explore the diverse experience of PwP and their caregivers. Despite the limitations of secondary data analysis, our data flag important unmet needs of PwP to be targeted in situations of confinement as similar lockdowns may reoccur during the current and future pandemics.

Our results will allow health professionals to expand their services in a meaningful way i.e., personalize their support in the identified themes and thus improve the health care of PwP in times of crisis. Future validation of the results seems reasonable to quantify and prioritize the identified self-perceived unmet needs. We recommend future research about unmet needs during confinement to assess caregiver burden, hospitalization and institutionalization in order to be able to understand the consequences of the unmet needs during pandemic.

## Data Availability Statement

The raw data supporting the conclusions of this article will be made available by the authors, without undue reservation. Requests to access the datasets should be directed to request.ncer-pd@uni.lu.

## Ethics Statement

The studies involving human participants were reviewed and approved by National Ethics Board (CNER Ref: 201407/13). The patients/participants provided their written informed consent to participate in this study.

## Author Contributions

A-MH: conception and design of the work, acquisition, analysis and interpretation of data, drafting, and final approval of the work. CP, LP, VS, and MH: acquisition, analysis and interpretation of data, drafting, critical revision, and final approval of the work. AK: acquisition of data, drafting, critical revision, and final approval of the work. GM, LH, and FW: acquisition of data, critical revision, and final approval of the work. RK: conception and design, critical revision, and final approval of the work. All authors agree to be accountable for all aspects of the work in ensuring that questions related to the accuracy or integrity of any part of the work are appropriately investigated and resolved.

## Conflict of Interest

The authors declare that the research was conducted in the absence of any commercial or financial relationships that could be construed as a potential conflict of interest.

## References

[1] Helmich RC Bloem BR The impact of the COVID-19 pandemic on Parkinson's disease: hidden sorrows and emerging opportunities. J Parkinsons Dis. (2020) 10:351–4. 10.3233/JPD-202038 32250324 PMC7242824

[2] Prasad S Holla VV Neeraja K Surisetti BK Kamble N Yadav R. Parkinson's disease and COVID-19: perceptions and implications in patients and caregivers. Mov Disord. (2020) 35:912–4. 10.1002/mds.28088 32304118 PMC7264599

[3] Schirinzi T Cerroni R Di Lazzaro G Liguori C Scalise S Bovenzi R. Self-reported needs of patients with Parkinson's disease during COVID-19 emergency in Italy. Neurol Sci. (2020) 41:1373–5. 10.1007/s10072-020-04442-1 32363506 PMC7196180

[4] Hipp G Vaillant M Diederich NJ Roomp K Satagopam VP Banda P. The Luxembourg Parkinson's study: a comprehensive approach for stratification and early diagnosis. Front Aging Neurosci. (2018) 10:326. 10.3389/fnagi.2018.00326 30420802 PMC6216083

[5] Tong A Sainsbury P Craig J. Consolidated criteria for reporting qualitative research (COREQ): a 32-item checklist for interviews and focus groups. Int J Qual Health Care. (2007) 19:349–57. 10.1093/intqhc/mzm042 17872937

[6] Vreman RA Heikkinen I Schuurman A Sapede C Garcia JL Hedberg N. Unmet medical need: an introduction to definitions and stakeholder perceptions. Value Health. (2019) 22:1275–82. 10.1016/j.jval.2019.07.007 31708064

[7] Deutsche Gesellschaft für Epidemiologie (DGEpi) Leitlinien und Empfehlungen zur Sicherung von Guter Epidemiologischer Praxis (GEP). Ulm: Deutsche Gesellschaft für Epidemiologie (DGEpi) (2018).

[8] Thomas J Harden A Methods for the thematic synthesis of qualitative research in systematic reviews. BMC Med Res Methodol. (2008) 8:45 10.1186/1471-2288-8-45 18616818 PMC2478656

[9] Polit DF Beck Tatano C. Nursing Research. Generating and Assessing Evidence for Nursing Practice. Philadelphia: Wolters Kluwers (2017).

[10] Miele G Straccia G Moccia M Leocani L Tedeschi G Bonavita S. Telemedicine in Parkinson's disease: how to ensure patient needs and continuity of care at the time of COVID-19 pandemic. Telemed J E Health. (2020) 26:1533–6. 10.1089/tmj.2020.0184 32667839

[11] Papa SM Brundin P Fung VSC Kang UJ Burn DJ Colosimo C. Impact of the COVID-19 pandemic on Parkinson's disease and movement disorders. Mov Disord. (2020) 35:711–5. 10.1002/mds.28067 32250460 PMC7996401

[12] Schenkman M Moore CG Kohrt WM Hall DA Delitto A Comella CL. Effect of high-intensity treadmill exercise on motor symptoms in patients with *de novo* Parkinson disease: a phase 2 randomized clinical trial. JAMA Neurol. (2018) 75:219–26. 10.1001/jamaneurol.2017.3517 29228079 PMC5838616

[13] van der Kolk NM de Vries NM Kessels RPC Joosten H Zwinderman AH Post B. Effectiveness of home-based and remotely supervised aerobic exercise in Parkinson's disease: a double-blind, randomised controlled trial. Lancet Neurol. (2019) 18:998–1008. 10.1016/S1474-4422(19)30285-6 31521532

[14] Radder DLM Sturkenboom IH van Nimwegen M Keus SH Bloem BR de Vries NM. Physical therapy and occupational therapy in Parkinson's disease. Int J Neurosci. (2017) 127:930–43. 10.1080/00207454.2016.1275617 28007002

[15] Mosley PE Moodie R Dissanayaka N. Caregiver burden in Parkinson disease: a critical review of recent literature. J Geriatr Psychiatry Neurol. (2017) 30:235–52. 10.1177/0891988717720302 28743212

[16] Peek CJ Baird MA Coleman E. Primary care for patient complexity, not only disease. Fam Syst Health. (2009) 27:287–302. 10.1037/a0018048 20047353

[17] Snoeck CJ Vaillant M Abdelrahman T Satagopam VP Turner JD Beaumont K Prevalence of SARS-CoV-2 infection in the Luxembourgish population: the CON-VINCE study. medRxiv [Preprint]. (2020). 10.1101/2020.05.11.20092916

