# Supplementary material for: Unmet Needs of People With Parkinson's Disease and Their Caregivers During COVID-19-Related Confinement: An Explorative Secondary Data Analysis
Source: Front Neurol. 2021 Jan 18;11:615172. doi: 10.3389/fneur.2020.615172 (PMC7848016; doi:10.3389/fneur.2020.615172)
Supplement: Supplementary file 1 [file Data_Sheet_1.pdf]

LUXEMBOURG INSTITUTE OF HEALTH
RESEARCH DEDICATED TO LIFE

Supplementary material

Supplementary material 1: Coding tree of the analytical themes related to unmet needs

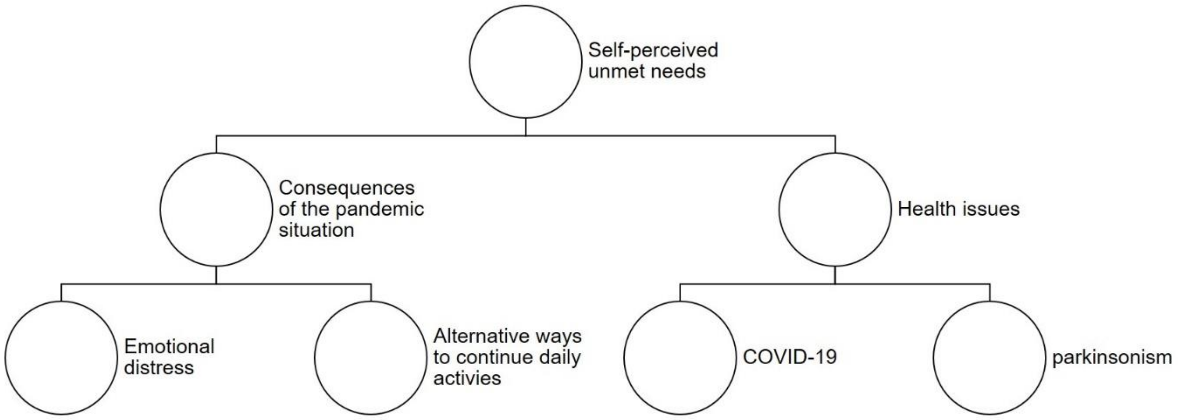

Supplementary material 2: Description of the analytical themes related to unmet needs

The following paragraph provides a description of the analytical themes. Given the multicultural context of Luxembourg, the interview notes were written in German, French or English. They were translated into English for illustration purposes.

1. Consequence of the pandemic situation: Emotional distress

Risk of infection with COVID-19 remains an important concern of PwP as the following note illustrates:

> *"Indirectly (he) let know about the fear of the virus. Hopes that it ends soon and that everyone will survive pandemic"* (Call 313).

But also the physical distancing and the restricted communication with family members, friends or everyday contacts were described as distressing:

> *"Patient is hospitalized because of an illness not related to COVID-19; wife feels isolated"* (Call 258).

PwP / Caregivers experienced non-adherence to hygiene recommendations by health care professions because of a lack of protection material (e.g. masks) and administrative issues (finances, insurance).

Indeed, caregivers and PwP reported the illness and / or death of a family member during confinement as an extreme emotional burden:

> *"Husband shows symptoms, Wife very anxious" (Call 547).*
>
> *"Wife is very ill (respiratory problems - negative for Corona) and PD patient struggles alone at home" (Call 105).*

2. Consequence of the pandemic situation: Alternative ways to continue daily activities

PwP and their caregivers needed to reschedule their private appointments, adapt to the new procedures in the transformed health system and cope with the new ways of communication with family and friends:

> *"Needs to see how to get his skin preparation. The dermatologist refused to send the prescription and told them that if they postponed the appointment they would have to wait. Will check with the pharmacy or their general practitioner" (Call 165).*
>
> *"Her husband says she's not getting better. A consultation was scheduled but cancelled due to the situation" (Call 100).*

Alternative ways to continue shopping and hobbies need to be explored:

> *"Nobody to help him for shopping. Didn't know if he was allowed to ask for a special home delivering service"* (Call 175)
>
> *"His wife is taking care of food supplies but she's already 80 years old and daughter is not coming often due to fear of corona"* (Call 23).
>
> *"Cleaning lady will bring groceries"* (Call 264).
>
> *" They don't need food or medication, but they would need incontinence material for the patient"* (Call 105).

3. Health Issues: COVID-19

As expected, unmet needs were also related to the general recommendations, the protection of vulnerable persons and procedures in case of contact with a COVID-19 positive person:

> *"Still manages to run his groceries on his own. Information given on precautions to be taken. Links sent by e-mail on the possibilities for the groceries. Mr is very happy with the solution, will call" (Call 166).*
>
> *"The mother of the wife (93yrs) is living with them and they try to reduce the risk of infection" (Call 657).*
>
> *"Asks how to deal if symptoms" (Call 412).*

COVID-19 symptoms were identified during the phone calls and participants were referred to a health professional:

> *"Spoke with the wife, they are both doing well, she had some respiratory symptoms, no testing, her husband (patient) didn't have symptoms"* (Call 299).

4. Health Issues: Parkinsonism

Questions related to the vulnerability of PwP, the interaction of PD-therapy with COVID-19, the availability of PD medication and the consequences of reduced physical activity were explored as important unmet needs related to Parkinsonism:

> *"Had questions about DBS and Corona (Interaction, more sensitive to the virus)" (Call 1).*
>
> *"Asked a lot of questions concerning delivery of duodopa pump medication. I told him that there is no problem about stock of medication" (Call 29).*
>
> *"Now the patient is stuck in Paris and he asks for a certificate stating that he has Parkinson's and a certificate certifying the need to go out and move around outside" (Call 135).*
>
> *"He's suffering from restricted movement due to staying at home" (Call 248).*

Unmet needs related to PD-related activities (Parkinson Association, Physiotherapy or the prescription of PD medication) remained essential during confinement:

> *"(...) he said that everything is ok but that he missed his kinesitherapy exercises" (Call 126).*
>
> *"Had to wait several days to receive his Prolopa at the pharmacy" (Call 424).*

Of note, self-perceived unmet needs unrelated to COVID-19 e.g. typical PD symptoms or side-effects of PD medication remained central:

> *"PD progresses, main problems are fatigue and falls" (Call 589).*
>
> *"Stopped Mirapexin but this caused sleep problems, went back to normal medication." (Call 209).*

Supplementary material 3: Description of the analytical themes related to the support network

The following paragraph describes the analytical themes more in detail.

1. Family

PwP and their caregivers reported their (vulnerable) partner, children (daughters and sons), grand-children, nephew and siblings being part of their support network during confinement:

> *"Patient feels good, no symptoms, no special needs, well surrounded by the family" (Call 89).*
>
> *"Patient is living alone but feels good at the moment, has no problems. She is in contact with her daughter if she needs anything" (Call 407).*
>
> *"Good surrounded by his husband who is currently working from home" (Call 416).*

2. Other support system

Cleaning staff, housekeeper, municipality, scouts or neighbors were part of the other support system as the following examples show:

> *"Cleaning lady will bring groceries (...)" (Call 264).*
>
> *"He is cared for by his daughter and a housekeeper who seems to live with him." (Call 497).*
>
> *"Municipality organizes food, medications, etc." (Call 209).*
>
> *"Patient is well looked after: Neighbour and friends have offered to do the shopping for them" (Call 359).*

3. Professional Care

Ambulant Nursing Service and nursing homes were named as professional support network of PwP and their caregivers:

> *"Ambulant nursing service comes once a day at home." (Call 607)*
>
> *"As she is staying in a nursing home. She is taken care of and does not require help." (Call 58)*

Some notes state that the patient had a good support system without specification.
